# Health-related quality of life and associated factors in people with diabetes at high risk of foot ulceration

**DOI:** 10.1186/s13047-022-00586-9

**Published:** 2022-11-18

**Authors:** Byron M. Perrin, Jaap J. van Netten, Wouter B. aan de Stegge, Tessa E. Busch-Westbroek, Sicco A. Bus

**Affiliations:** 1 La Trobe Rural Health School, La Trobe University, PO Box 199, Bendigo, Victoria 3552 Australia; 2 Amsterdam UMC, location University of Amsterdam, Rehabilitation Medicine, Meibergdreef 9, Amsterdam, The Netherlands; 3 Amsterdam Movement Sciences, Rehabilitation and Development, Amsterdam, The Netherlands

**Keywords:** Diabetic foot, Ulcer recurrence, Quality of life, Health surveys

## Abstract

**Background:**

The health-related quality of life (HRQoL) of people with diabetes-related foot complications has been increasingly reported, mostly from studies of people with a foot ulcer. The aim of this study was to assess HRQoL and determine factors associated with HRQoL in people with diabetes at high risk of foot ulceration.

**Methods:**

In all, 304 participants enrolled in the Diabetic Foot Temperature Trial (DIATEMP) were included in the cross-sectional analysis. HRQoL was measured by the RAND® 36-Item Short Form Health Survey (SF-36) at baseline. Potential factors associated with HRQoL were analysed using multiple linear regression analyses for the eight domains of the SF-36.

**Results:**

Participants were predominantly male (72%), mean age 64.6 (±10.5) years, 77% type 2 diabetes and mean duration of diabetes 20 (±14) years. Mean SF-36 domain scores for the General Health (49.2 ± 20.1), Role Physical (50.9 ± 44.7), Physical Function (58.5 ± 27.9) and Vitality domains (59.8 ± 21.6) were lower compared to the Mental Health (78.4 ± 18.0), Social Functioning (75.3 ± 24.2), Role Emotional (73.5 ± 38.9) and Bodily Pain (67.0 ± 27.0) domains. HRQoL was lower than Dutch population-based and general diabetes samples, but higher than in samples with an ulcer. Use of a walking aid was associated with lower HRQoL across all 8 SF-36 domains (β range − 0.20 to − 0.50), non-Caucasian descent was associated with lower HRQoL in 5 domains (β range − 0.13 to − 0.17). Not working, higher BMI and younger age were associated with lower HRQoL in 3 domains.

**Conclusions:**

People at high risk of diabetes-related foot ulceration have reduced HRQoL that varies across domains, with the physical domains most affected. Assessing mobility, ethnicity, BMI and job status may be useful in daily practice to screen for people who might benefit from interventions targeting HRQoL.

**Trial registration:**

Netherlands Trial Registration: NTR5403. Registered on 8 September 2015.

## Background

The International Diabetes Federation has estimated that 463 million people had diabetes in 2019, and 1 in 10 people (693 million) will have diabetes by 2045 [1]. People with diabetes are at high risk of developing lower-extremity complications, including peripheral neuropathy and peripheral artery disease, which can lead to foot ulceration and lower-extremity amputation [2]. Recent research has shown that diabetes-related foot complications are a large and growing contributor to the disability burden worldwide, with an estimated 2.1% of the global years lived with disability (YLD), and 59% of all diabetes-related YLD [3]. Diabetes-related foot complications are hard to manage and often recur, especially in the case of ulceration [4].

Health-related quality of life (HRQoL) and other patient-reported outcomes provide additional information to traditional biomedical measures of health when assessing preventative interventions or outcomes of treatment [5]. Over the past two decades, the HRQoL of people with diabetes-related foot complications has been increasingly investigated [5]. These studies predominantly focus on people with diabetes-related foot ulceration and a recent meta-analysis has shown that the HRQoL of people with such foot ulcers is low, especially with respect to physical function and perceptions of general health [6]. HRQoL has hardly been investigated in those people who heal from an ulcer. More insight in this population is important, because of the high risk for ulcer recurrence, [4] and the association between HRQoL and worsening foot morbidity [7].

There is also limited data on the factors associated with HRQoL in people at highest risk of ulceration [8]. Understanding the factors associated with HRQoL in this population may assist with identifying key areas of intervention that can impact patients’ HRQoL. Such insights could assist in the design of screening programs and development of personalised or behavioural interventions to promote well-being and contribute to ulcer prevention [9]. Thus, the aim of this study was to assess HRQoL in people with diabetes at high risk of foot ulceration and to determine factors associated with HRQoL.

## Methods

### Study design

This was a cross-sectional analysis of HRQoL data obtained at baseline from people enrolled in the Diabetic Foot Temperature Trial (DIATEMP), a multi-centre randomised controlled trial that aimed to assess the cost-effectiveness and cost utility of at-home foot temperature monitoring to reduce the incidence of foot ulcer recurrence [10, 11].

### Participants

Informed consent was obtained by participants enrolled from multi-disciplinary clinics and affiliated podiatry clinics of three university and four community hospitals spread across the Netherlands. All participants were at high risk of developing a diabetes-related foot ulcer, with all having diabetes, loss of protective sensation and a history of foot ulceration (within the past 4 years) or a history of Charcot neuro-osteoarthropathy [10, 11]. Loss of protective sensation was measured using a 10-g Semmes-Weinstein monofilament and a 128 Hz tuning fork and was diagnosed if participants failed one or both tests. Participants were excluded if, at the time of assessment, they had a foot ulcer, foot infection, an active Charcot neuro-osteoarthropathy, critical limb ischaemia, bilateral amputation proximal to the tarso-metatarsal (Lisfranc) joint, an estimated survival of less than 18 months, or if they were already using home temperature monitoring as part of their foot care. Further details on the inclusion and exclusion criteria is described elsewhere [10, 11].

### Outcomes of interest

#### Health-related quality of life as measured by SF-36

Health-related quality of life was measured with the Dutch translation of the RAND® 36-Item Short Form Health Survey (SF-36) (Version 1.0) [12, 13]. The survey was administered at baseline in the participating setting. The SF-36 is a comprehensive, standardised and psychometrically sound generic measure of health status in general and specific populations [12, 14]. The SF-36 includes one scale for each of eight important domains of health: Physical Function (practical physical activity), Role Physical limitations (physical health-related role limitations), Role Emotional (accomplishment of work or other usual activities), Vitality (levels of energy), Mental Health (feelings of peace, happiness and calmness), Social Functioning (health-related physical or emotional effects on the quantity and quality of social activities), Bodily Pain and General Health (measuring a general rating of health and the views and expectations of health) [12]. Details on the survey and item responses can be found elsewhere [12–14].

#### Potential factors associated with HRQoL

Participant characteristics measured at baseline in the DIATEMP trial were considered as potential factors associated with HRQoL [10]. These include variables according to setting (university medical centre, community hospital, podiatry practice); general demographics (age, sex, ethnicity, education levels, if living alone, if working; socio-economic score measured by postcode linked to the Netherlands Institute for Social Research); diabetes-related variables (diabetes type, duration, control, BMI); presence of microvascular complications such as retinopathy, nephropathy or need for dialysis; health-related behaviour (smoking, alcohol consumption); use of a walking aid to improve mobility (walking stick, walking frame, wheelchair, scooter); footwear (including walking barefoot at home and use of orthopaedic footwear); and foot health at baseline (presence of peripheral artery disease [15], presence of foot deformity [16], presence of minor foot skin lesions, history of lower-extremity amputation, time since healing of previous foot ulcer and duration of previous two ulcers).

### Statistical analysis

The SF-36 was scored using a two-step process [12, 13]. First, numeric values were recoded so that a higher score defined a more favourable health state and were in a 0 (worst possible) to 100 (best possible) range. Outcome data was normally distributed so items in the same scale were averaged together to create eight mean health domain scale scores. Items left blank by respondents were not considered, so the final domain score represented the average for all items in a scale that the participants answered [13]. For all other independent variables, consolidation of groups was undertaken where appropriate for a regression analysis. To determine the factors associated with HRQoL, multiple linear regression analyses were used for each of the eight SF-36 health domains with statistically significant (*p* < 0.200) independent variables found after univariate analysis entered into the multivariate models [17]. Factors associated with HRQoL for each health domain of the SF-36 were determined if statistically significant (*p* < 0.05) after multivariate analysis. The analysis was performed with SPSS, version 26 (SPSS Inc., Chicago, IL).

## Results

### Participant characteristics and HRQoL

A total 304 participants were recruited from the participating centres with 29% recruited from a university medical centre, 44% from a community hospital and 27% from podiatry practice. Participant characteristics are detailed in Table 1. Mean age was 64.6 ± 10.5 years, with 72.4% men and mean duration of diabetes 20 ± 14 years. Two hundred and ninety-five participants were included based on history of ulceration and nine participants were included based on having a history of Charcot neuro-osteoarthropathy.

**Table 1:** Participant characteristics (*N* = 304)

Characteristic	All	Missing
Setting		
University medical centre	29 (88)	
Community hospital	44 (134)	
Podiatry practice	27 (82)	
Age (years)	64.6 ± 10.5	
Sex		
Male	72 (220)	
Female	28 (84)	
Ethnicity		
Caucasian	93 (283)	
Non-Caucasian	6.9 (21)	
Education		0.7 (2)
Low or medium	70 (213)	
High	29 (89)	
Living status		
Live alone	35 (105)	
Not live alone	65 (199)	
Work status		
Working	25 (75)	
Not Working	75 (229)	
Socio-Economic Score	−0.24 ± 1.2	1.0 (3)
Type of diabetes		1.3 (4)
Type 1	22 (66)	
Type 2	77.0 (234)	
Years diagnosed with diabetes	20 ± 14	1.0 (3)
Glycated haemoglobin (mmol/mol)	60.7 ± 16.0	21 (65)
Body mass index (kg/m2)	29.8 ± 5.3	0.3 (1)
History of microvascular complications	55 (166)	1.0 (3)
Smoking or history of smoking	56 (169)	
> 2 units alcohol intake per day	66 (199)	
Use of walking aid	29 (89)	
Footwear		0.7 (2)
Conventional	32 (97)	
Semi or full orthopaedic shoes	67 (205)	
Walk barefoot at home	37 (113)	
Peripheral artery disease		
Grade 1	65 (197)	
Grade 2	35 (107)	
Presence of foot deformity^a^	94 (287)	
Presence of Minor lesions^b^	40 (121)	10 (31)
History of amputation^c^	27 (81)	
Months between healing of most recent ulcer and study entry	10.1 ± 11.1	3.3 (10)^d^
Months duration of last 2 previous ulcers	8.8 ± 28.6	0.3 (1)

*Note*: Data are expressed as % (n) or mean ± SD

^a^Foot deformity was classified into one of four categories according to the severity of deformity present [10]

^b^Minor lesion defined as a haemorrhage, blister, abundant callus, or erythema, identified at entry

^c^In case of bilateral amputation, the highest level was chosen

^d^Including 9 participants that were included based on having a history of Charcot neuro-osteoarthropathy

Mean SF-36 domain scores are found in Table 2. Lowest scores were found for General Health (49.2 ± 20.1), while highest scores were found for Mental Health (78.4 ± 18.0). The mean score for Role-Physical (50.9 ± 44.7) was next lowest, with Physical Function (58.5 ± 27.9), Vitality (59.8 ± 21.6) and Bodily Pain (67 ± 27). Along with Mental Health, Social Functioning (75.3 ± 24.2) and Role-Emotional (73.5 ± 38.9) had a mean score > 70.

**Table 2:** Mean SF36 domain scores (*n* = 304)

SF-36 domain	Mean ± SD	% missing
Physical Function	58.5 ± 27.9	4.3
Role Physical	50.9 ± 44.7	4.3
Role Emotional	73.5 ± 38.9	5.0
Vitality	59.8 ± 21.6	4.3
Mental Health	78.4 ± 18.0	4.3
Social Functioning	75.3 ± 24.2	4.0
Bodily Pain	67.0 ± 27.0	4.6
General Health	49.2 ± 20.1	4.0

### Factors associated with HRQoL

Factors identified after univariate analyses  were determined (Table 3) and entered into the multivariate analyses and the significant factors associated with the eight domains of the SF-36 are presented in Table 4. There were nine factors associated with one or more of the eight SF-36 domains. Of these, use of a walking aid (29% of the study sample) was associated with each of the 8 domains of HRQoL, where those using a walking aid reported lower mean domain scores than those who did not. This difference was most marked in the Physical Functioning, Role Physical and Bodily Pain domains. Caucasian descent (93% of the sample) was also a prominent factor of HRQoL, where those identifying as Caucasian had significantly higher and more favourable scores in the Role Physical, Role Emotional, Mental Health, Social Functioning and Bodily Pain domains. Older age was associated with higher Vitality, Mental Health and General Health domain scores. Those working (25% of sample) had higher Physical Function, Vitality and General Health domain scores and. A higher BMI was associated with lower Physical Function, Vitality and Bodily Pain scores. Alcohol intake was associated with higher Role Physical score. Those with type 2 diabetes (77% of sample) had a higher Social Functioning score compared to those with type 1 diabetes. There were two foot-health related factors associated with HRQoL, with those with minor lesions (40% of sample) reporting higher Vitality and Social Functioning scores than those without and those with a history of amputation (27% of sample) reporting higher Bodily Pain mean scores than those without.

**Table 3:** Factors entered in the multivariate models for each of the SF-36 domains^a^

**Physical Function**	**Role Physical **	**Role Emotional **	**Vitality**	**Mental Health **	**Social Functioning **	**Bodily Pain **	**General Health**
Age (r = -.15, p = 0.010) Sex (t = 4.9,p < 0.001) Education (t = -2.1, p = 0.035) Work (t = 5.7, p < 0.001) SES score (r = .12, p = 0.040) Walking aid (t = -13.1,p < 0.001) Orthopedic shoes (t = 1.3, p = 0.191) Barefoot (t = -1.8, p = 0.078) BMI (r = -.27, p < 0.001) Alcohol (t = -2.8, p = 0.005)	Sex (t = 3.3, p = 0.001) Ethnicity (t = -2.3, p = 0.024) Education (t = -1.9, p = 0.065) Work (t = 2.8, p < 0.01) SES score (r = .1, p = 0.069) Walking aid (t = -7.4,p < 0.001) BMI (r = -.12, p = 0.040) Alcohol (t = -2.8, p = 0.005) Smoking (t = 2.0, p = 0.042) Minor lesions (t = -1.6, ,p = 0.104)	Ethnicity (t = -2.1, p = 0.047) Education (t = -1.3, p = 0.184) Work (t = -2.9, p = 0.005) SES score (r = 0.1, p = 0.198) Walking aid (t = -3.5,p < 0.001) Smoking (t = -1.5, p = 0.147) Alcohol (t = -1.8, p = 0.073) Minor lesions (t = -1.4, p = 0.177) Microvascularcomplications (t = -1.4, p = 0.074)	Age (r = .12^.^p = 0.039) Sex (t = -2.6, p = 0.010) Ethnicity (t = 2.4, p = 0.016) Work (t = 2.1, p = 0.033) SES score (r = 0.1, p = 0.192) Walking aid (t = -5.1,p < 0.001) BMI (r = -.18, p < 0.003) Alcohol (t = -2.7, p < 0.007) Minor lesions (t = 2.2, p = 0.029)	Age (r = .15, p = 0.009) Sex (t = -1.6, p = 0.111) Ethnicity (t = -2.3, p = 0.035) Work (t = -1.8, p = 0.073) Live alone (t = 1.7, p = 0.095) Walking aid (t = -4.0,p < 0.001) BMI (r = -.1, p = 0.124) Alcohol (t = 2.6, p = 0.006) Minor lesions (t = -1.9, p = 0.063)	Sex (t = 3.6,p < 0.001) Ethnicity (t = -2.4, p = 0.016) Work (t = 2.7, p < 0.01) SES score (r = 0.1, p = 0.195) Walking aid (t = -6.1,p < 0.001) Diabetes type (t = -2.5, p = 0.011) Diabetes duration (r = -.1, p = 0.059) BMI (r = -.1, p = 0.080) Alcohol (t = 2.7, p < 0.008) Minor lesions (t = 2.9, p < 0.01)	Sex (t = 4.0,p < 0.001) Ethnicity (t = -2.0, p = 0.048) Education (t = -2.1, p = 0.033) Work (t = 4.1,p < 0.001) Walking aid (t = -7.8,p < 0.001) Barefoot (t = -1.4,p = 0.161) BMI (r = -.18, p = 0.002) Alcohol intake (t = -3.2, p = 0.002) Amputation (t = 2.7, p = 0.007) Minor lesions (t = -1.8, p = 0.078) Time ulcer heal (r = -.1, p = 0.129)	Age (r = .22, p < 0.001) Sex (t = -1.8, p = 0.087) Ethnicity (t = -2.3, p = 0.024) Work (t = 1.8, p = 0.070) Live alone (t = 1.4, p = 0.150) Diabetes type (t = -1.5, p = 0.134) Diabetes Duration (r = -.1, p = 0.100) BMI (r = -.1, p = 0.081) Alcohol (t = -1.9, p = 0.062) Walking aid (t = -4.1,p < 0.001) Foot deformity (t = 1.7, p = 0.092) HB1Ac (r = -.18, p = 0.005) Microvascular complications (t = 2.1, p = 0.034)

^a^Each of the potential factors with HRQOL were individually analysed for each of the eight SF-36 scales using Pearson’s r correlation for ratio data or independent samples t-test for dichotomous data

**Table 4:** Factors associated with SF-36 domains after multivariate analysis

**SF36 Domain**	**Age**	**Ethnicity**	**Work**	**Walking Aid**	**Alcohol**	**Diabetes Type**	**BMI**	**Minor Lesions**	**Amputation**
**Physical Function** R^2^=0.46, p < 0.001			β = .15, p < 0.01 Working: 73.90 ± 23.3 Not working: 53.4 ± 27.4	β = -.50, p < 0.001 Walking aid: 31.5 ± 21.8 No walking aid: 69.1 ± 22.3			β = -.14, p < 0.01		
**Role Physical** R^2^=0.26, p < 0.001		β = -.13, p = 0.020 Caucasian: 52.5 ± 44.5 Non-Caucasian: 28.5 ± 42.3		β = -.37, p < 0.001 Walking aid: 22.9 ± 38.5 No walking aid: 62.3 ± 42.1	β = .17, p = 0.005 Alcohol intake: 58.7 ± 43.5 No Alcohol intake: 36.3 ± 43.5				
**Role Emotional** R^2^=0.12, p < 0.001		β = -.17, p = 0.007 Caucasian: 75.0 ± 37.7 Non-Caucasian: 50.0 ± 48.9)		β = -.20, p = 0.002 Walking aid: 61.0 ± 43.5 No walking aid: 78.5 ± 35.7					
**Vitality** R^2^=0.17, p < 0.001	β = .14, p = 0.034		β = .15, p = 0.023 Working: 64.4 ± 20.3 Not working: 58.2 ± 21.9	β = -.23, p < 0.001 Walking aid: 50.1 ± 21.3 No walking aid: 63.7 ± 20.5			β = -.13, p = 0.030	β = .14, p = 0.018 Minor lesions: 62.6 ± 19.4 No minor lesions: 56.8 ± 22.9	
**Mental Health** R^2^=0.15, p < 0.001	β = .16, p = 0.014	β = -.17, p = 0.006 Caucasian: 79.3 ± 16.9 Non-Caucasian: 65.8 ± 26.3		β = -.20, p = 0.002 Walking aid: 71.9 ± 19.4 No walking aid: 81.0 ± 16.7					
**Social Functioning** R^2^=0.22, p < 0.001		β = -.13, p = 0.022 Caucasian: 76.2 ± 23.6 Non-Caucasian: 62.8 ± 29.4		β = -.31, p < 0.001 Walking aid: 62.3 ± 25.7 No walking aid: 80.5 ± 21.6		β = .24, p = 0.006 Type 2 diabetes: 77.4 ± 21.6 Type 1 diabetes: 68.6 ± 30.9		β = .13, p = 0.026 Minor lesions: 79.4 ± 21.5 No minor lesions: 70.8 ± 25.8	
**Bodily Pain** R^2^=0.32, p < 0.001		β = -.14, p = 0.015 Caucasian: 67.8 ± 26.8 Non-Caucasian: 55.5 ± 26.4		β = -.40, p < 0.001 Walking aid: 49.3 ± 26.1 No walking aid: 74.2 ± 23.8			β = -.13, p = 0.023		β = .19, p = 0.001 Prior amputation: 73.4 ± 23.0 No prior amputation: 64.6 ± 27.9
**General Health** R^2^=0.22, p < 0.001	β = .34, p < 0.001		β = .21, p = 0.003 Working: 52.8 ± 21.5 Not working: 47.9 ± 19.5	β = -.21, p = 0.002 Walking aid: 41.8 ± 17.3 No walking aid: 52.1 ±20.4					

Whilst all multivariate models were significant, the largest explanatory effect (46%) was seen for Physical Function domain scores (R^2^ = 0.46), based on use of walking aid, working status and BMI. The smallest explanatory effect (12%) was seen for the Role Emotional domain scores (R^2^ = 0.12), based on ethnicity and use of a walking aid.

## Discussion

### Health-related quality of life of people at high risk of diabetes-related ulceration

**Fig. 1:**
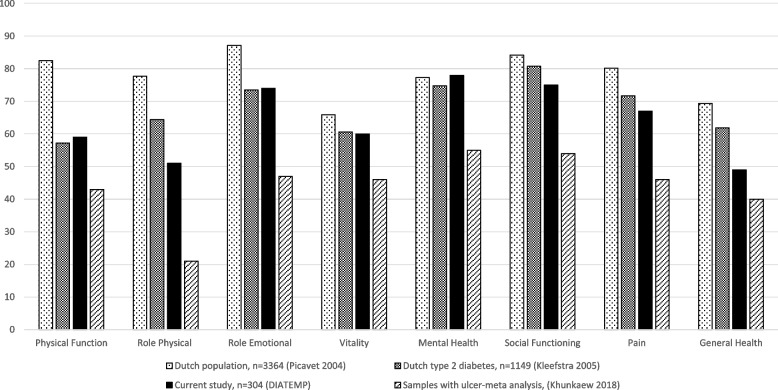
DIATEMP baseline SF36 scores compared to selected studies from different samples

This is the first study of HRQoL from a large sample of people at high risk of diabetes-related foot ulceration. Overall, the most significant HRQoL impairment was in the General Health, Role Physical and Physical Function domains. The Mental Health domain scored highest, followed by Social Functioning, Role Emotional, Bodily Pain and Vitality. In comparison to other populations, the HRQoL of our study sample at high risk of ulceration was lower than of representative Dutch population-based [18] and diabetes [19] samples, but higher than in samples with a current ulcer [6] (see Fig. 1 for a visual depiction of this pattern). This trend has also been found in previous studies in people with diabetes-related foot complications such as ulceration, infection or amputation, indicating HRQoL is lower when groups have worse foot and ulcer outcomes [5]. This also suggests that the protective benefits to the HRQoL of people remaining in ulcer remission may be significant, especially across physical functioning and general health.

The reported impairments in Physical Function and Role Physical are understandable given the population studied. All participants in this study had peripheral neuropathy, and it has been shown that people with peripheral neuropathy exhibit symptoms such as unsteadiness or limitations to balance which can influence falls risk [20]. Limitations to Physical Function and Role Physical have also been found in a small Dutch cross-sectional study of people with ulceration assessing physical mobility, where the potential was raised for HRQoL to be increased with rehabilitation interventions to enhance mobility [21]. It is also understandable that the General Health domain was low in the current study. Participants reported a mean score over twenty points lower than a Dutch general population [18], and a reduction in the General Health domain is a consistent finding amongst people with diabetes [22].

There were more favourable HRQoL scores reported across the mental-emotional-social domains of the SF-36. The Mental Health domain had the highest mean domain score, which is a similar finding in samples with a foot ulcer [5, 6, 8]. It is important though to not discount the role peripheral neuropathy has on mental health in light of clear associations being found between peripheral neuropathy with anxiety and depression, and it is possible that the mental and emotional domains of the SF-36 do not adequately capture neuropathy-related emotional distress [23]. There was also a comparatively high domain score for Role Emotional and Social Functioning, with scores consistently higher than reported in people with ulceration [6]. Vitality was lower compared to the Mental Health and Role Emotional scores, and this reduction in energy levels can also be seen in the general and other populations (Fig. 1).

### Factors associated with health-related quality of life

The independent variables entered into the regression analyses explained between 12 to 46% of the variance in the eight SF-36 domain scores. The strongest model showed that working status, use of walking aid and BMI combined predicted 46% of the variance in the Physical Function domain, with not working, having a walking aid and a higher BMI independently associated with lower Physical Function scores. Overall, the factors associated with HRQoL concerned mainly individual and lifestyle characteristics (e.g. age, ethnicity, working status, walking aid, alcohol intake), while, with the exception of BMI, there were fewer diabetes- or foot health-related factors associated with HRQoL. Key areas of clinical focus should include addressing potentially modifiable factors associated with HRQoL such as interventions to reduce the need for a walking aid, to lower BMI and to improve employment potential.

The use of a walking aid was the strongest associated factor of HRQoL across all domains of the SF-36. While this is a new finding, use of a walking aid has recently been found from the DIATEMP sample to be a predictor of plantar foot ulceration [24]. For the 29% of the participants who used a walking aid, scores indicated lower HRQoL compared to non-users of walking aids across both physical and mental domains of HRQoL, with the largest effect being for Physical Function and Role Physical. Walking aids can indicate disability, mobility problems, balance impairment and risk of falling [25]. However, as the design for this study was a randomised controlled trial on the effectiveness of foot thermometry, there were limited variables collected and analysed relating to using a walking aid such as balance and safety [25]. However, these findings suggest that assessment of physical function might be useful in people at high risk of foot ulceration to identify areas of important interventions to improve HRQoL. This could include the prescription of rehabilitation programs [21] that include exercise training, as adherence to exercise has been shown to be associated with better HRQoL in the Physical Function, Vitality, Mental Health, Social Functioning and Bodily Pain domains [8].

Participant ethnic and racial background was also a consistent factor of HRQoL, with Caucasians scoring more favourably than non-Caucasians on Role Physical, Role Emotional, Mental Health, Social Functioning and Bodily Pain domains. Although caution must be taken when interpreting these results as there were only 7% non-Caucasian participants in the study, it is significant that the non-Caucasian participants reported lower HRQoL across these domains. This may reflect a level of disadvantage and a need for additional support for their care. However, selection bias is a possibility, as most of the non-Caucasian participants attended a university medical centre and had higher morbidity (data not shown). Further studies including larger non-Caucasian populations remain needed to investigate this association.

The next most prevalent factors associated with lower HRQoL were younger age (in domains Vitality, Mental Health, General Health), work status (Physical Function, Vitality, General Health) and higher BMI (Physical Function, Vitality, Bodily Pain). Work status as a factor of HRQoL is consistent with research in people with diabetes showing that paid work is associated with lower risk of impairment in activities of daily living and provides valuable social participation [26]. As has been found in other clinical populations of people with history of ulceration [27], the younger participants in this study were more likely to have type 1 diabetes and worse blood glucose control (data not shown), indicating worse morbidity with possible subsequent effect on HRQoL. Similarly, the negative association between BMI with HRQoL is consistent with the literature [28].

There are few studies that have specifically investigated the factors associated with HRQoL in people with or at risk of diabetes-related foot ulceration. Our findings contrast with previous findings from a sample of people with ulceration, where the key factors associated with HRQoL as measured by the SF-36 were biochemical signs of inflammation [29]. Two other previous studies of people with healed ulceration have investigated factors associated with self-perceived health [30, 31]. One found that younger age, cardiovascular disease and depression were associated with poorer self-perceived health [30], contrasting with a more recent study that showed older age, female gender, low education, high BMI and presence of complications were associated with poorer perceived health [31]. These contrasting results from different populations suggest that further research is required to explore the potentially different factors associated with HRQoL for people with different levels of diabetes-related foot morbidity.

### Methodological considerations

This study from a relatively large and representative sample adds information that is currently lacking about the HRQoL in people at high risk of ulceration. The data appears valid when observing the pattern of results compared to previous research across different populations, as they align with data that suggests worsening morbidity is associated with worse HRQoL (Fig. 1). The design of the DIATEMP trial, where the focus of the study was on incident ulceration and foot thermometry, limited the measurement of other potentially relevant HRQoL parameters, such as other co-morbidities, medication regime, and more detailed sociodemographic information. This may be reflected in the relatively low predictive power of the multivariate analyses. Further, whilst the SF-36 is an established generic measure of HRQoL, it has been asserted that it may inadequately measure mental and emotional responses in this population [5, 23].

## Conclusions

This study has determined the HRQoL in a large sample of people at high risk of diabetes-related foot ulceration. People at high ulcer risk have reduced HRQoL across most domains of HRQoL in comparison to the general population, and higher than in studies reporting on people with diabetes and a foot ulcer. Use of walking aid was associated with reduced HRQoL across all domains of the SF-36. Non-Caucasian ethnicity, not working, higher BMI and younger age were the next most common factors associated with reduced HRQoL. Although causal inferences cannot be made from this study, assessing mobility, ethnicity, BMI and job status may be useful in daily practice to screen for people who might benefit from interventions targeting HRQoL. Future research should investigate the clinical implications of these association over time and to explore possible causal pathways.

## Data Availability

The datasets used and/or analysed during the current study are available from the corresponding author on reasonable request.

## Abbreviations

HRQoL: Health-related quality of life
DIATEMP: Diabetic Foot Temperature Trial
BMI: Body Mass Index
YLD: Years lived with disability
SF-36: RAND® 36-Item Short Form Health Survey (Version 1.0)
